# Exploring the relationship between air pollution and meteorological conditions in China under environmental governance

**DOI:** 10.1038/s41598-020-71338-7

**Published:** 2020-09-03

**Authors:** Yansui Liu, Yang Zhou, Jiaxin Lu

**Affiliations:** 1 Institute of Geographic Sciences and Natural Resources Research, Chinese Academy of Sciences, 11A Datun Road, Chaoyang District, Beijing, 100101 China; 2 Key Laboratory of Regional Sustainable Development Modeling, Chinese Academy of Sciences, Beijing, 100101 China

**Keywords:** Atmospheric science, Environmental impact

## Abstract

Extensive studies have been carried out on the impact of human activities on air pollution, but systematic investigation on the relationship between air pollutant and meteorological conditions is still insufficient, especially in the context of China’s site scale and recent comprehensive environmental pollution control. Here, we used a spatial interpolation technology to establish a set of data sets of pollutants and meteorological elements that are spatially matched at 896 stations in China to reveal the air pollutant-meteorological interactions between 2014 and 2019. We found that air pollution and meteorological elements have obvious seasonal and regional characteristics. Over the last few years, the concentration of most air pollutants in China has dropped significantly except for O_3_. The increase in O_3_ concentration was closely related to the decrease of particulate matter and NO_2_ concentration. The concentration of most air pollutants was affected by meteorological conditions, but the level of impact depended on the type of pollutants and varied across regions. The concentration of air pollutants at most stations was significantly negatively correlated with wind speed, precipitation and relative humidity, but positively correlated with atmospheric pressure. As the latitude increases, the impact of temperature on the concentration of air pollutants becomes more obvious. To effectively control air pollution, it is further urgent to reveal the relationship between air pollution and meteorological conditions based on long-term daily or real-time data.

## Introduction

Intense human activities and adverse meteorological conditions worsen air quality and damage human health^1–4^. Air pollution has multiple health effects, such as increasing the incidence of cardiovascular and respiratory disorders^5–9^, diabetes and hypertension^10^, dementia^11^ and the abortion risk for pregnancies^3^, and causing psychiatric and mental^12^ and premature deaths^2^, and impairing memory^13^ and affecting peoples’ cognitive ability^14^ and reducing life expectancy^15,16^. Serious air pollution can also increase criminal and unethical behavior^17^, lower urbanites’ happiness^18^, decrease solar power potential^19^ and cause huge economic damages^20^.

With the rapid urbanization and industrialization over the past three decades, China has become one of few countries with the most severe air pollution in the world. Especially in January 2013, severe haze blanketed Beijing and northern China, affecting more than 600 million people^21^. In 2013, the PM_2.5_ level of 58 cities in China was five times higher than the safety level set by the World Health Organization (WHO), which has attracted worldwide attention. Extensive studies have focused on the causes^22–25^, spatiotemporal changes and driving forces of air pollution^21,26,27^, and its impact on social economy and public health in China^6,28^. Most studies focus on heavily polluted areas in China, such as 31 provincial capitals^29^, North China Plain^30^, Yangtze River Delta^31^ and Sichuan Basin^32^. The consensus is that China's air quality has improved significantly in recent years due to strong political commitment to fight against air pollution and comprehensive emission control^33–36^. For example, between 2013 and 2017, PM_2.5_ concentration in 74 cities in China has been reduced by 33% through target-driven emission reduction regulation^37^. These findings have provided a useful reference for understanding the spatio-temporal characteristics of air pollution in China and for policy-makers to formulate energy-saving and pollution-reducing policies.

In addition to the strong impact of human activities, adverse weather conditions are also considered to be one of the main reasons for China's severe air pollution in recent years^33,38–41^. Meteorological conditions can explain more than 70% of the variance of pollutant concentrations in China’s 31 provincial capital cities^33^. Among meteorological conditions, temperature and wind speed are generally considered to be two major factors affecting the concentration of air pollutants in China^42^. For example, frequent severe haze episodes in Beijing are considered to be closely relate to adverse meteorological conditions^43^. Furthermore, regional transportation of pollutants with wind is also an important way to cause severe haze pollution in China^44^. Undoubtedly, meteorological conditions have an important influence on the formation of air pollution and changes in pollutant concentration. A systematic understanding on the relationship between air pollutant concentration and meteorological conditions is the prerequisite and basis for scientifically formulating air pollution prevention and control policies. Although existing studies have tried to explore the relationship between air pollution and meteorological conditions at a few sites or typical areas, there is still a lack of systematic investigations at the national site scale, especially in the context of China's efforts to comprehensively control environmental pollution in recent years. Based on the real-time data of six air pollutant concentrations (PM_2.5_, PM_10_, CO, SO_2_, NO_2_ and O_3_) and daily meteorological data (average temperature, wind speed, atmospheric pressure and relative humidity, accumulated precipitation at 20–20) from June 2014 to February 2019, this study applied partial correlation analysis, multivariable linear regression (MLR) and panel data models to investigate the spatio-temporal characteristics of air pollutant concentration and meteorological factors at the site scale in China and the relationship between the two in the last five years.

## Results

### Spatio-temporal characteristics of air pollutants and meteorological factors

**Figure 1:**
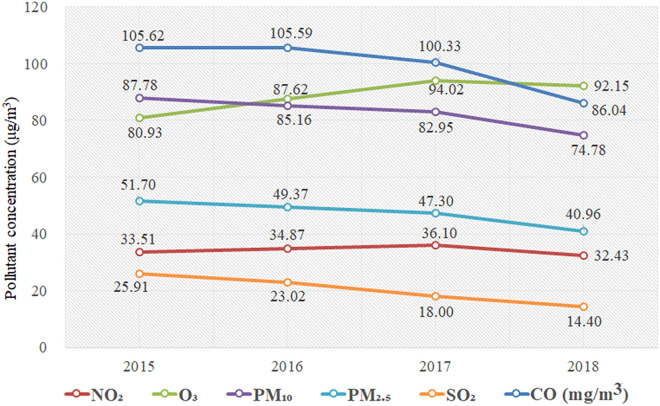
Annual variation of air pollutant concentration in China between 2015 and 2018.

In the past few years, China's air quality has improved significantly. Between 2015 and 2018, the concentrations of most pollutants showed a downward trend except for O_3_ (Fig. 1). Compared with 2015, the concentrations of SO_2_, PM_2.5_, PM_10_, NO_2_ and CO in China decreased by 44%, 21%, 15%, 3% and 19% respectively by 2018. The average concentration of SO_2_ decreased from 25.91 µg/m^3^ in 2015 to 14.4 µg/m^3^ in 2018, with an average annual decrease of 4 µg/m^3^. The average annual concentrations of PM_2.5_ and PM_10_ have decreased significantly from 51.7 ug/m^3^ and 87.78 ug/m^3 ^in 2015 to 40.96 ug/m^3^ and 74.78 ug/m^3^ in 2018, respectively, with an average annual decrease of 3.4 µg/m^3^ and 4.1 µg/m^3^. Obviously, the concentration of most air pollutants in China has decreased significantly in recent years, but O_3_ concentration is on the rise. The average annual concentration of O_3_ has increased from 80.93 µg/m^3^ in 2015 to 92.15 µg/m^3^ in 2018, with an average annual increase rate of 4%.

**Figure 2:**
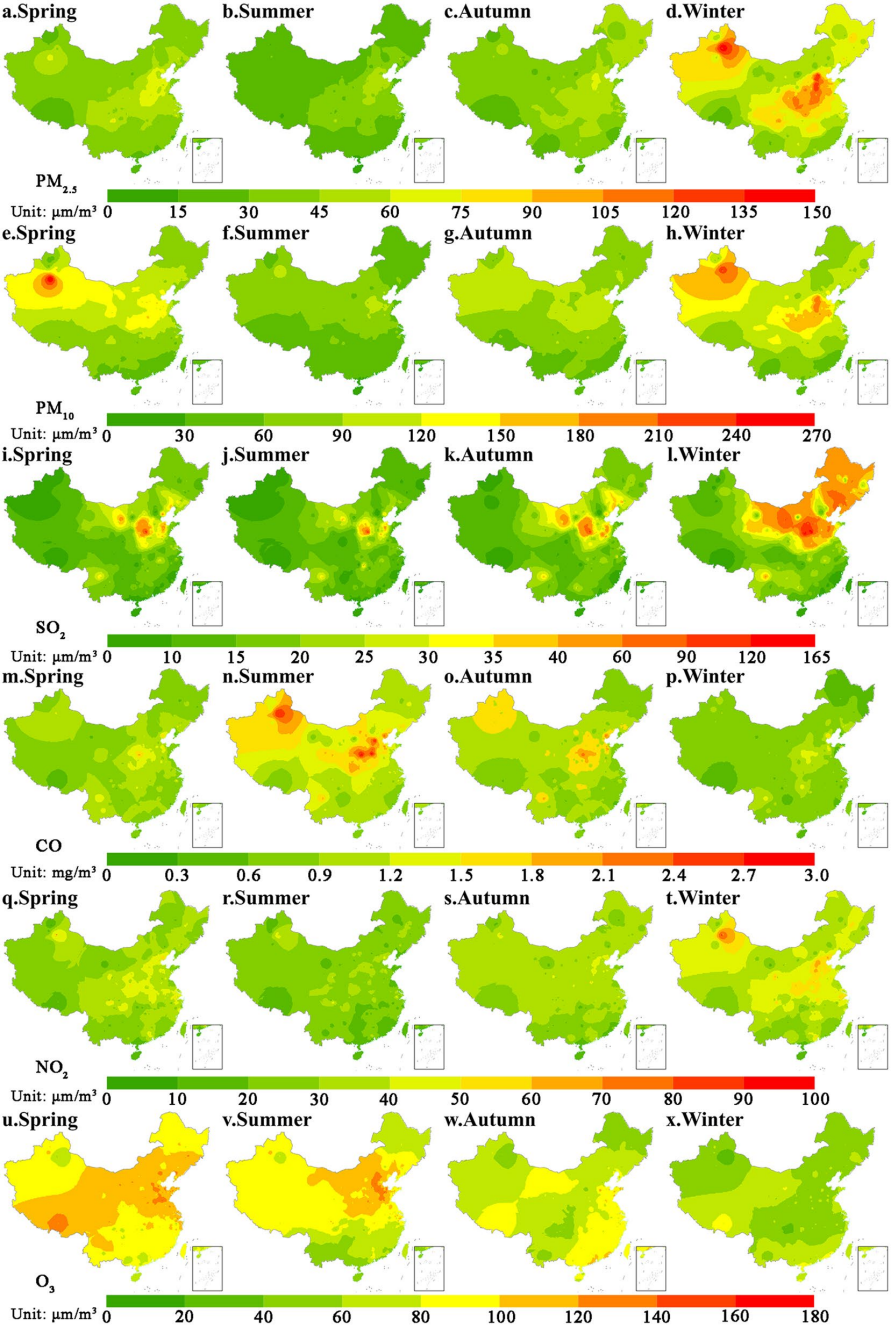
Seasonal variation of six air pollutant concentrations between 2015 and 2018. (Notes: The map was generated using ESRI’s ArcGIS 10.2 (https://desktop.arcgis.com/en/arcmap); The data was obtained by spatial interpolation of the seasonal values of six air pollutant concentrations at 896 air quality observation stations across the country).

Air pollution in China showed obvious seasonal and regional characteristics (Fig. 2). The concentrations of PM_2.5_, PM_10_, SO_2_ and NO_2_ were high in winter and low in spring. The areas with high concentrations of PM_2.5_, PM_10_ and NO_2_ were mainly concentrated in the North China Plain and Xinjiang, while that of SO_2_ were concentrated in the North China Plain, the Northeast Plain and the Inner Mongolia Plateau. The high concentrations of PM_2.5_ and PM_10_ in the North China Plain may be related to the intense human activities and unfavorable meteorological conditions, while that in Xinjiang may be caused by frequent sandstorms^34^. The concentration of CO was high in summer and autumn. Northern China was a high concentration area of CO, especially the North China Plain and Northwest China. Surprisingly, the concentration of O_3_ and other pollutants presented distinct seasonal and regional characteristics. The O_3_ concentration in spring and summer was significantly higher than that in autumn and winter. The Qinghai-Tibet Plateau, North China Plain, Northeast Plain and Inner Mongolia Plateau have higher O_3_ concentrations in spring.

**Figure 3:**
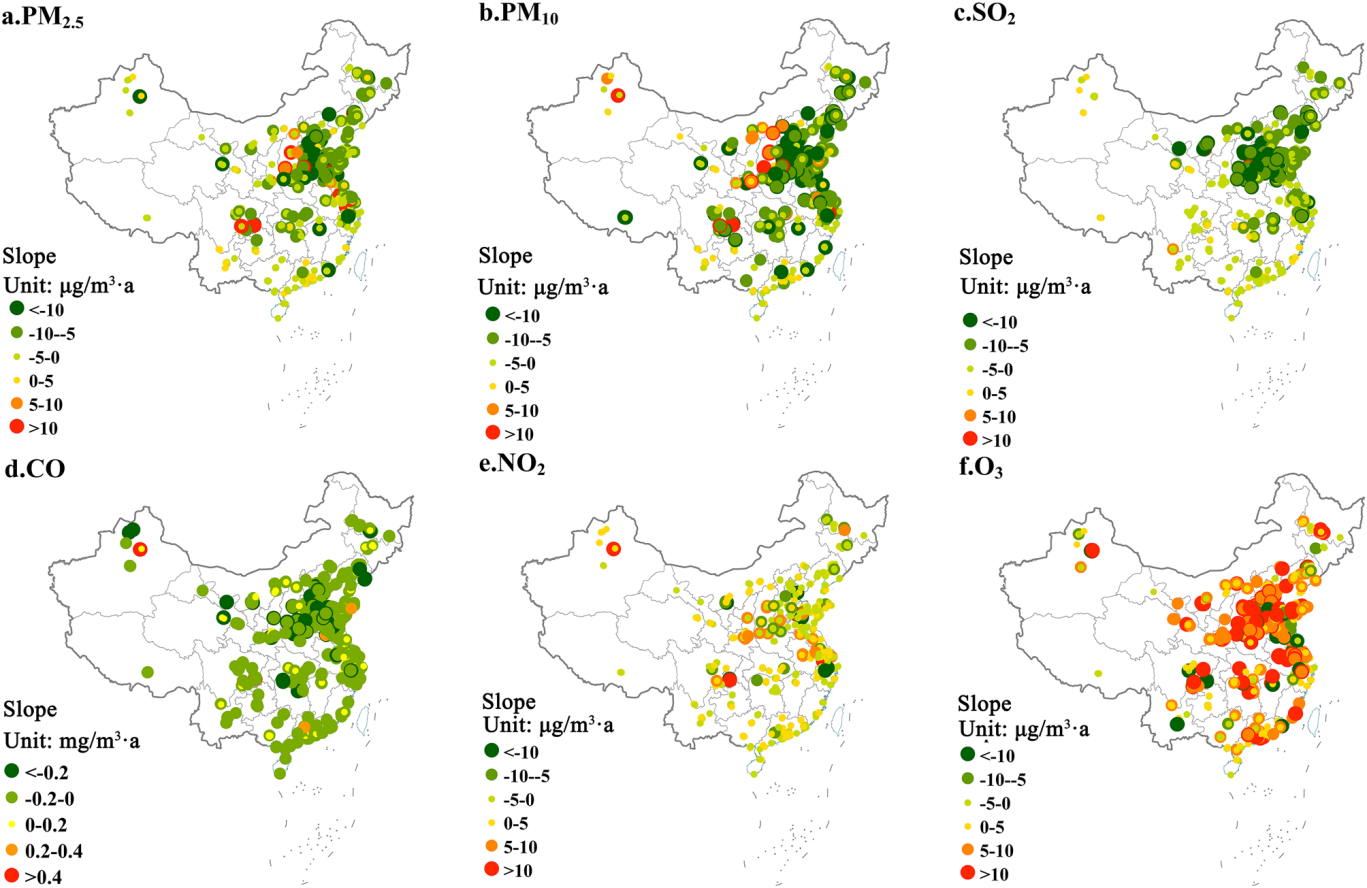
Trend changes of six air pollutant concentrations at 896 stations in China from 2015 to 2018. (Notes: The map was generated using ESRI’s ArcGIS 10.2 (https://desktop.arcgis.com/en/arcmap); A positive slope indicates the increase of pollutant concentration, and vice versa. The greater the absolute value of the slope is, the faster the pollutant concentration rises or falls).

Figure 3 shows the change trend of air pollutant concentrations at 896 stations across the country from 2015 to 2018. Results indicate that in addition to O_3_, concentration of other air pollutants at more than two-thirds of the sites across the country showed an obvious downward trend. The spatial variation of PM_2.5_ and PM_10_ concentrations presented basically the same characteristics. Among the 896 sites in this study, the PM_2.5_ concentration at 53 sites and the PM_10_ at 122 sites decreased at an average annual rate of more than 10 µg/m^3^ per year. Like particulate matter, between 2015 and 2018, the annual average CO concentration of more than 80% stations also showed a downward trend. The concentration of SO_2_ has dropped more significantly, with the concentration of more than 92% of the sites in the country showing a downward trend. On the contrary, the O_3_ concentration of more than 80% of the sites across the country has shown a rapid upward trend, and most sites have an average annual increase of 5 µg/m^3^ per year.

**Figure 4:**
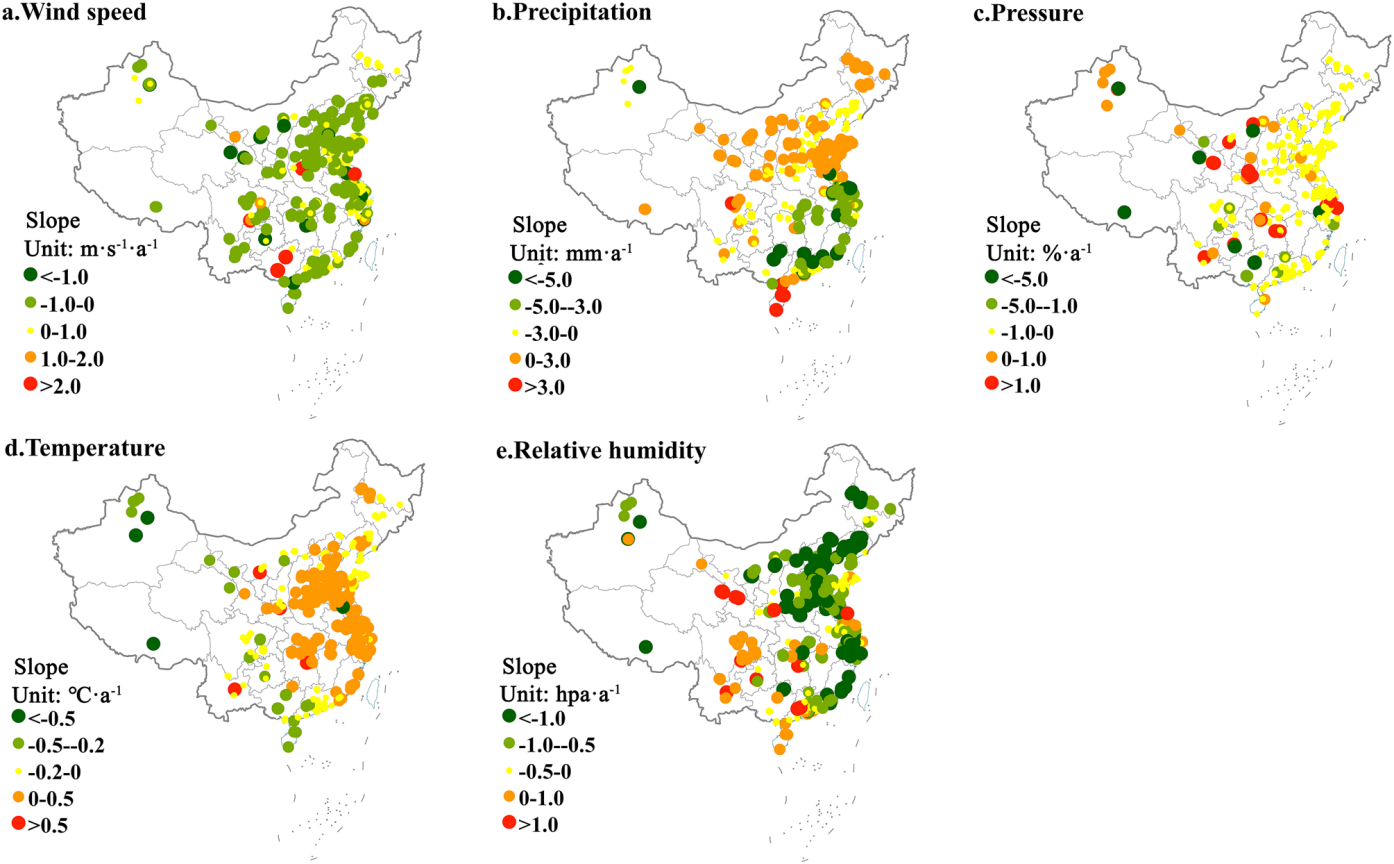
Variation trends of meteorological elements at 896 stations nationwide from 2015 to 2018 (Notes: The map was generated using ESRI’s ArcGIS 10.2 (https://desktop.arcgis.com/en/arcmap); A positive slope indicates the increase of meteorological elements (such as wind speed, temperature, precipitation, relatively humidity and atmospheric pressure), and vice versa. The greater the absolute value of the slope is, the faster the meteorological factor rises or falls).

We also investigated the changing trend of five meteorological factors at 896 stations in China from 2015 to 2018 (Fig. 4). The results demonstrate that the annual average wind speed, relative humidity and atmospheric pressure in most stations showed a downward trend. In recent years, the eastern and central regions demonstrated a warming trend, while the northwest region exhibited a cooling trend. The average annual wind speed of 69% of the stations in the whole country showed a downward trend. The average wind speed of 285 stations in China displayed an increasing trend, mainly distributed in the eastern coast, northeast Heilongjiang Province and a small part of the Yangtze River Basin. The average annual precipitation decreased in the south and increased in the north, but showed a downward trend. The average annual precipitation of more than 65% of the stations in China demonstrated a downward trend. In addition, the average annual atmospheric pressure at about 90% of the stations in China also showed a downward trend.

### The pollutant-meteorological interactions

#### Correlation between air pollutant concentration and meteorological factors

Based on the monthly average data (June 2014–February 2019) of 896 stations, Partial correlation analysis was used to explore the relationship between air pollutant concentration and meteorological conditions in China. The results suggest that most of the air pollutant concentrations were negatively correlated with wind speed, precipitation and relative humidity, but positively correlated with atmospheric pressure (Table 1). For the whole year, the concentrations of the six pollutants were significantly negatively correlated with precipitation and relative humidity with wind speed held constant. The partial-correlation coefficients between atmosphere pressure and NO_2_, O_3_, PM_2.5_ and PM_10_ concentration with wind speed held constant were 0.084, 0.100, 0.164 and 0.078, respectively, which means that the increase in atmospheric pressure will increase the concentration of air pollutants. When the precipitation is kept constant, relative humidity and wind speed were significantly negatively correlated with the concentrations of most air pollutants, but positively correlated with atmospheric pressure. Seasonally, the concentration of most pollutants was significantly negatively correlated with wind speed, precipitation and relative humidity, but positively correlated with atmospheric pressure. However, in addition to O_3_, the absolute values of partial correlation coefficients between PM_2.5_, PM_10_, CO, SO_2_, NO_2_ concentration and meteorological conditions in autumn and winter were significantly higher than that in spring and summer (Supplementary Table S1). This result indicates that the changes in air pollutant concentrations in autumn and winter were greatly affected by meteorological conditions.

**Table 1:** Partial **c**orrelation coefficient between air pollutant concentration and meteorological factors in China based on 896 stations.

Control variable	Variable	CO	NO_2_	O_3_	PM_2.5_	PM_10_	SO_2_
WS	Pre	− 0.435***	− 0.217***	− 0.103***	− 0.418***	− 0.601***	− 0.371***
	AP	− 0.102	0.084**	0.100***	0.164***	0.078**	− 0.105
	Tem	− 0.272***	− 0.171***	− 0.004	− 0.251***	− 0.406***	− 0.309***
	RH	− 0.468***	− 0.223***	− 0.164***	− 0.300***	− 0.532***	− 0.409***
Tem	RH	− 0.424***	− 0.146***	− 0.247***	− 0.173***	− 0.375***	− 0.283***
	AP	− 0.002	0.162***	0.145***	0.283***	0.100***	0.039
	WS	− 0.120***	− 0.083**	0.178***	− 0.138***	− 0.121***	0.008
	Pre	− 0.402***	− 0.144***	− 0.163***	− 0.402***	− 0.528***	− 0.215***
Pre	RH	− 0.187***	− 0.063*	− 0.163***	0.151	− 0.014	− 0.191***
	AP	0.059*	0.174***	0.170***	0.359***	0.187***	0.048
	WS	− 0.145***	− 0.079**	− 0.161***	− 0.167***	− 0.147***	0.028
	Tem	0.220***	0.041	0.102***	0.238***	0.255***	− 0.002
RH	Pre	− 0.059*	− 0.046	0.072**	− 0.324***	− 0.323***	− 0.037
	AP	0.112***	0.197***	0.218***	0.325***	0.193***	0.101***
	WS	− 0.164***	− 0.084**	0.145	− 0.133***	− 0.129***	0.011
	Tem	0.197***	0.023***	0.157***	0.002	0.042	0.013
AP	Pre	− 0.408***	− 0.254***	− 0.198***	− 0.492***	− 0.605***	− 0.378***
	RH	− 0.447***	− 0.272***	− 0.279***	− 0.389***	− 0.543***	− 0.424***
	WS	− 0.021	− 0.026	0.189	− 0.055*	0.034	0.125
	Tem	− 0.225***	− 0.205***	− 0.124***	− 0.315***	− 0.396***	− 0.318***

Obs. is 896. WS, Pre, RH, Tem and AP indicate annual average value of wind speed, precipitation, relatively humidity, temperature and atmosphere pressure, respectively. *, ** and *** indicate statistical significance at the 10%, 5% and 1% levels, respectively.

The correlation between air pollutant concentration and meteorological conditions at the site scale in China was displayed in Supplementary Fig. S1. The results indicate that except for O_3_, the concentration of air pollutants at most sites was significantly negatively correlated with wind speed, precipitation and relative humidity, but significantly positively correlated with atmospheric pressure. The O_3_ concentration at most sites was significantly positively correlated with wind speed and precipitation, but negatively correlated with atmospheric pressure. The PM_2.5_ concentration at more than 70% of sites was significantly negatively correlated with wind speed, precipitation and relative humidity, but significantly positively correlated with atmospheric pressure. The PM2.5 concentration of a few sites in the Beijing-Tianjin-Hebei region and the Shandong Peninsula was significantly positively correlated with wind speed, which may be related to the topographical conditions of these areas. For example, the concentration of air pollutants in the southern part of Beijing is significantly higher than that in the northern area. This is mainly because the northwest wind in Beijing gradually weakened as the distance increased, transporting pollution from the north to the south, thereby increasing the concentration of pollutants in the south. For O_3_, in addition to atmospheric pressure and relative humidity, its concentration at most sites was significantly positively correlated with wind speed, precipitation and temperature. O_3_ concentration and relative humidity had a significant positive correlation with relative humidity in the eastern coastal areas, but a significant negative correlation in the central and western regions.

### Impact of meteorological conditions on air pollutant concentration

**Figure 5:**
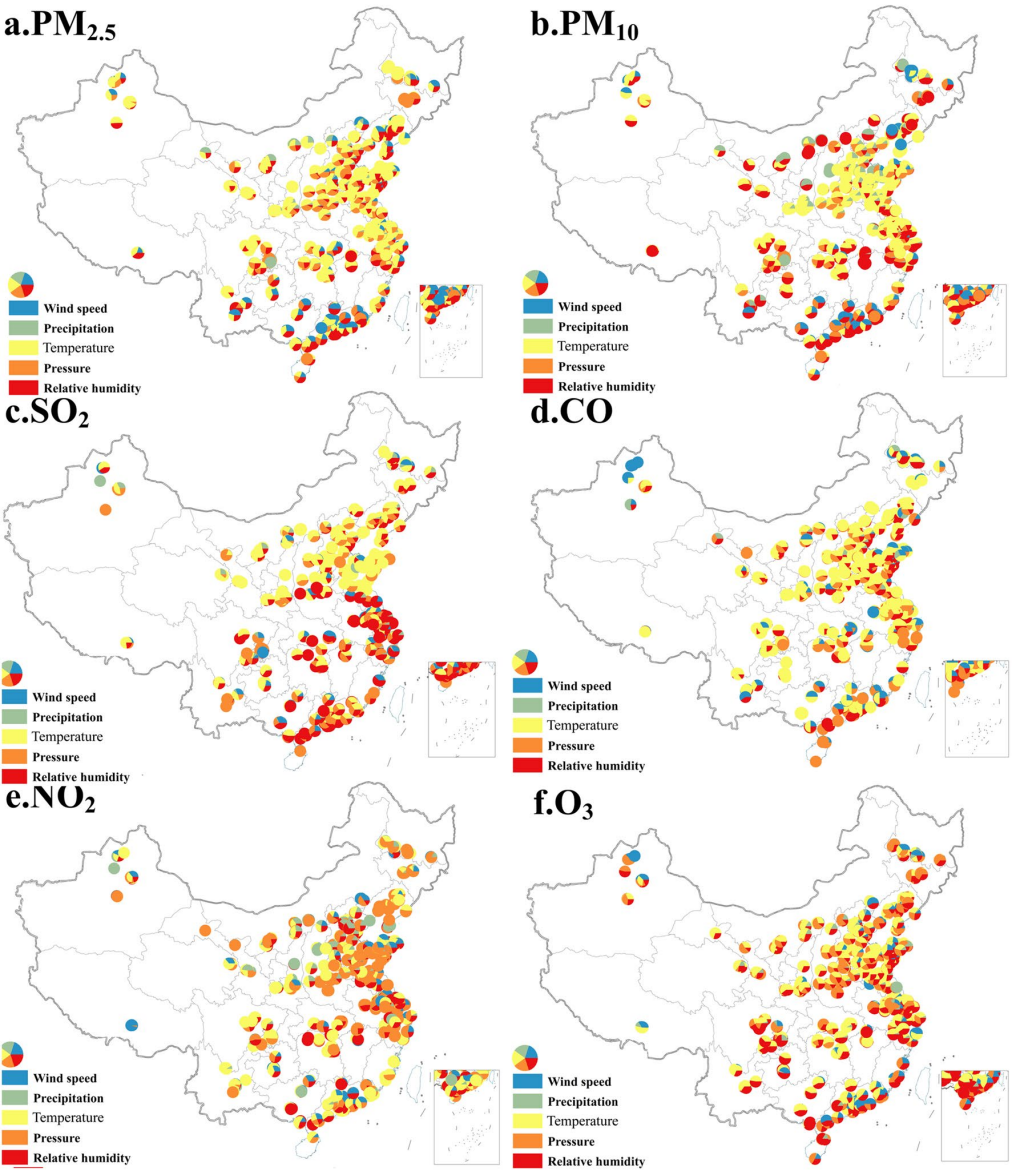
The estimated results of MLR model for 896 sites in China. (Notes: The map was generated using ESRI’s ArcGIS 10.2 (https://desktop.arcgis.com/en/arcmap); In the MLR model, the independent variables are average wind speed, average temperature, precipitation, relative humidity, and average atmospheric pressure, and the dependent variable is the concentration of six air pollutants. The data used in the model includes the monthly value of pollutant concentration and meteorological elements of 896 stations from June 2014 to February 2019 (57 months in total). The F-test was used to test the significance of the model, and the t-test was used to test the significance of independent variables and *R*^2^ to judge the fitting effect of the model. Among the 896 regression models of PM_2.5_, PM_10_, SO_2_, CO, NO_2_ and O_3_ concentrations in China, respectively, the significance of the MLR model of 848, 840, 675, 778, 788 and 862 sites passed the *F*-test and at least one independent variable passed the t-test at the 10% significance level or higher. *R*^2^ of the models ranged from 0.19 to 0.93 and its fitting effect was good. The five colors in the pie chart respectively represent the meteorological elements that affect the concentration changes of various pollutants at each station. The marked influencing factors pass the significance level test of at least 10%. The proportion of each color in the pie chart represents the absolute value of the regression coefficient of each meteorological element. The larger the value, the greater the proportion of the corresponding color in the pie chart, indicating the greater the contribution of the meteorological element to the change of pollutant concentration.).

MLR model was used to further identify the main meteorological factors affecting pollutant concentrations at each site. Results demonstrate that the concentration of air pollutants at most sites was affected by multiple meteorological factors, but the magnitude of the impact depended on the type of pollutants and varied across regions. For PM_2.5_, among the 848 sites where the model passed the *F*-test and at least one independent variable passed the *t*-test, the number of sites with an estimated coefficient of wind speed, precipitation, atmospheric pressure, temperature, and relative humidity to PM_2.5_ concentration less than zero was 614, 650, 514, 835 and 470, respectively. These results indicate that the PM_2.5_ concentration at most sites was affected by meteorological conditions, and the improvement of meteorological conditions can greatly reduce PM_2.5_ concentration. As the altitude increases, the contribution of temperature to the reduction of PM_2.5_ concentration gradually increases, while that of wind speed and relative humidity decreases. The meteorological conditions that affect the concentration of air pollutants in Northeast China were also distinct. For example, the PM_2.5_ concentration of most sites in Liaoning Province was mainly affected by temperature, relative humidity and wind speed, while that was affected by temperature and wind speed in the southwest of Heilongjiang (Fig. 5a).

For PM_10_, of the 896 sites, the significance of MLR model at 840 sites passed the *F*-test, and at least one independent variable passed the *t*-test. Among the 840 sites, the number of sites with an estimated coefficient of wind speed, precipitation, atmospheric pressure, temperature, and relative humidity to PM_2.5_ concentration less than zero was 598, 641, 457, 780 and 676, respectively. The influence of meteorological conditions on PM_10_ concentration also had obvious regional differences. In the North China Plain, temperature was recognized as the dominant factor affecting PM_10_ concentration, while wind speed and relative humidity as the dominant factors in Northeast China. Except for the North China Plain and the central and southern regions, relative humidity was identified as the dominant factor for PM_10_ concentration changes at most sites in China (Fig. 5b).

For SO_2_, among the 675 sites where the significance of the MLR model passed the *F*-test and at least one independent variable passed *t*-test, the number of sites with an estimated coefficient of less than zero for wind speed, precipitation, atmospheric pressure, temperature, and relative humidity to SO_2_ concentration was 411, 326, 381, 519 and 437, respectively. This result indicates that the change in SO_2_ concentration at 61%, 48%, 56%, 77% and 65% of sites was significantly negatively correlated with wind speed, precipitation, atmospheric pressure, temperature and relative humidity, respectively. Compared with particulate matters (PM_2.5_ and PM_10_), the number of stations where weather conditions have a significant impact on SO_2_ concentration has reduced by 20%. In the area south of the Yangtze River Basin, SO_2_ concentration at most sites was significantly negatively correlated with relative humidity. In Northern China, temperature was the dominant factor affecting SO_2_ concentration (Fig. 5c). For CO, among the 778 sites where the model passed the *F*-test and at least one independent variable passed the *t*-test, the number of sites with an estimated coefficient of less than zero for wind speed, precipitation, atmospheric pressure, temperature and relative humidity to CO concentration was 456, 494, 501, 736 and 238, respectively. Temperature has been identified as the dominant factor in CO concentration changes at most sites. The sites where wind speed has a significant impact on CO concentration were mainly distributed in southern China, Shandong peninsula and northern Xinjiang. Relative humidity had also been identified as one of the main meteorological influencing factors of CO concentration changes in the eastern coastal areas of China (Fig. 5d). For NO_2_, in addition to temperature, atmospheric pressure has also been identified as one of the leading meteorological factors for changes in pollutant concentration in the eastern coastal and northeastern regions of China. Relative humidity also had a significant impact on NO_2_ concentration in the Beijing-Tianjin-Hebei region (Fig. 5e). As far as O_3_ is concerned, the meteorological conditions of more than 96% of sites across the country have a significant impact on its concentration. Temperature, relative humidity, and atmospheric pressure were identified as the dominant factors for pollutant concentration changes at most sites. The increase in O_3_ concentration at more than 92% sites was mainly due to the increase in temperature and the decrease in relative humidity. The increase in O_3_ concentration in 73% of sites nationwide was related to the decrease in atmospheric pressure (Fig. 5f).

The estimated results from panel data model further demonstrate that most air pollutant concentrations were significantly negatively correlated with wind speed, temperature, precipitation, and relative humidity (Table 2). The concentration of PM_2.5_, PM_10_, SO_2_ and NO_2_ decreased by 0.16, 0.22, 0.12 and 0.12 µg/m^3^ for every 1 m/s increase in average wind speed, respectively. The increase of temperature had a significant and negative effect on SO_2_ concentration, but a positive effect on O_3_ concentration. The concentration of SO_2_ decreased by 0.85 µg/m^3^ for every 1 °C increase in the average temperature. The O_3_ concentration increased by 2.63 µg/m^3^ for every 1 °C increase in the average temperature. In addition, the increase in atmospheric pressure had a positive effect on the increase of PM_2.5_, PM_10_, and NO_2_ concentration, but had a negative effect on O_3_, CO, and SO_2_.

**Table 2:** Estimated results of impact of meteorological elements on six air pollutant concentrations in China.

Variable	PM_2.5_	PM_10_	CO	NO_2_	O_3_	SO_2_
**Unstandardized estimated coefficient**						
WS	− 0.155***	− 0.220***	− 0.002***	− 0.122***	0.365***	− 0.116***
Pre	− 0.083***	− 0.097***	0.000	− 0.049***	− 0.089***	0.042***
AP	0.053***	0.139***	− 0.001***	0.138***	− 0.289***	− 0.056**
Tem	− 1.429***	− 1.486***	− 0.023***	− 0.519***	2.626***	− 0.849***
RA	0.087***	− 0.587***	0.003***	− 0.001	− 0.813***	− 0.181***
**Standardized estimated coefficient**						
WS	− 0.044***	− 0.040***	− 0.031***	− 0.060***	0.078***	− 0.036***
Pre	− 0.090***	− 0.068***	0.004	− 0.093***	− 0.073***	0.050***
AP	0.107***	0.180***	− 0.131***	0.486***	− 0.439***	− 0.125**
Tem	− 0.541***	− 0.362***	− 0.478***	− 0.342***	0.747***	− 0.354***
RA	0.041***	− 0.178***	0.066***	− 0.001	− 0.288***	− 0.094***
Constant	18.287*	19.190	2.364***	− 88.613***	380.263***	103.085***
Obs	51,072	51,072	51,072	51,072	51,072	51,072
*R*^2^	0.424	0.345	0.253	0.342	0.507	0.174
Sites	896	896	896	896	896	896

Estimated result is based on the equilibrium panel data through the cross-section fixed effect model. The number of cross sections is 896 and the time series is 57 months (from June 2014 to February 2019). WS, Pre, RH, Tem and AP indicate annual average value of wind speed, precipitation, relatively humidity, temperature and atmosphere pressure, respectively. Constant is the constant term. *, ** and *** indicate statistical significance at the 10%, 5% and 1% levels, respectively.

### Discussion

Favorable meteorological conditions and joint efforts to save energy and reduce emissions have greatly reduced the concentration of air pollutants in China^45^. To mitigate air pollution, the Chinese government issued the first National Action Plan for Air Pollution Prevention and Control (2013–2017) in 2013, which requires that by 2017, the concentration of PM_10_ in cities and above prefecture level be reduced by over 10% compared with the 2012 level^21^. To realize this goal, the country has actively cracked down on high-emission and technologically outdated factories and vehicles, and promoted shifts from coal to gas in residential heating and the upgrading of industrial structure and energy efficiency improvement. During the period 2010–2018, although China’s primary energy consumption continued to increase, the growth rate of most provinces showed a significant downward trend, and the energy consumption intensity is also decreasing. These measures had led to cuts in PM_10_, SO_2_ and NO_x_ by 22.7%, 59% and 21%, respectively, during the period 2013–2017. To further improve air quality, the Chinese government implemented a new action plan for clean air in 2018. The new plan sets a goal of reducing the total emissions of sulfur dioxide, nitrogen oxides and volatile organic compounds by 15%, 15% and 10% compared with 2015 by 2020, respectively^46^.

In the past few years, with the implementation of powerful comprehensive control measures for air pollution, the concentration of air pollutants in China has dropped significantly. In addition to the increase in O_3_ concentration, the concentration of air pollutants at most stations across the country has fallen, with the most significant decrease in the Beijing–Tianjin–Hebei region. This is not only due to the improvement of meteorological conditions in the region, but also the result of coordinated prevention and control of air pollution and reduction in residential fuel emissions in recent years^47–49^. The increase in O_3_ concentration may be due to the changes in anthropogenic emissions that significantly improve air quality in terms of PM_2.5_ levels, led to a change in the ratio of chemicals in the air and an intensification of ozone pollution^50,51^. We found that except for a few sites in the Pearl River Delta, the O_3_ concentration of most other sites in China was significantly and negatively correlated with PM_2.5_, PM_10_, and NO_2_ concentration (Supplementary Fig. S2). This indicates that the decrease of particulate matter and NO_2_ concentration may cause the increase of O_3_ concentration. Achieving a coordinated control of PM_2.5_ and O_3_ concentration will be one of the biggest challenges for China’s next phase of air-cleaning campaign.

Although the concentration of pollutants at most sites has dropped significantly, further air pollution control still faces severe challenges. The concentration level of most pollutants was still higher than the health standard set by the WHO. By the end of 2018, the concentrations of PM_2.5_ and PM_10_ were lower or close to the national safety level of 35 µg/m^3^ and 75 µg/m^3^ set by China’s class two regions, but they are still far higher than the safety level of 10 µg/m^3^ and 20 µg/m^3^ set by the WHO. At the site scale, about 61% (554) and 42% (382) of the sites respectively exceeded the China’s national standards in terms of PM_2.5_ and PM_10_ concentration, and almost all stations exceeded the WHO’s safety level. In 2018, the country’s 8-h ozone concentration was 92.15 µg/m^3^, which is far lower than the national level of 160 µg/m^3^ in two classes regions in China and slightly lower than the safety level of 100 µg/m^3^ of the WHO, but there is still more than one-third of the sites in the country whose ozone concentration exceeds the safety level set by WHO. Air pollution control is not an overnight matter, and it is affected by many factors such as human activities, geographical environmental conditions, and social and economic development levels^23,33,52^.

Favorable weather conditions contribute to the diffusion of air pollutant concentrations and improve air quality. The influence of meteorological conditions on air pollutant concentration depends on the type of pollutant and the degree of influence varies across regions. Except for O_3_, the concentration of other air pollutants (such as PM_2.5_, PM_10_, SO_2_, CO, NO_2_) at most sites in China was significantly and negatively correlated with average wind speed, precipitation and relative humidity, but positively correlated with atmospheric pressure. Although the concentration of air pollutants in most stations was affected by multiple meteorological factors, the degree of impact depended on the type of pollutants and the geographical location of the stations. These results are consistent with previous studies, and their findings indicated that an increase in wind speed is beneficial to improve air quality^8,33^. However, we detected that the increase in temperature helps reduce the concentration of air pollutants. This is inconsistent with previous studies, which found a positive correlation between temperature and pollutant concentration^33,53^. This may be related to the monthly data used in this study to reveal the relationship between temperature and pollutant concentration. The analysis based on monthly data may not reflect the relationship between temperature and air pollutant concentration in real time, resulting in a certain degree of uncertainty. Therefore, combining the intensity of human activities and environmental governance measures, the relationship between air pollutants and meteorological conditions based on daily or real-time observation data deserves further research.

## Methods

The data used in this study include the hourly data of atmospheric pollutant observation stations and the daily data of meteorological stations during the period from June 1, 2014 to February 28, 2019. Air pollutants include PM_2.5_, PM_10_, SO_2_, CO, NO_2_ and O_3_ (O_3__8h), and their concentration data comes from China’s National Environmental Monitoring Platform (NEMP) (https://www.aqistudy.cn). Since January 2013, the NEMP began to publish real-time hourly concentrations of PM_2.5_, PM_10_, SO_2_, CO, NO_2_ and O_3_ based on the 896 monitoring stations in 190 cities of China. By May 2014, the number of air quality monitoring stations increased to 946, and by January 2015, to 1,436. This study draws on Barrero et al.’s *Z*-score standardization method to control the quality of air pollution data. First, the time series of hourly average concentrations were standardized using the *Z*-score standardization, and then the outliers were excluded^33,54^. In addition, sites with discontinuous pollutant concentration data or missing values over 10 days were also excluded. Finally, hourly data on the concentration of six air pollutants in 896 air quality observation stations across the country was obtained. The daily meteorological data, including average wind speed, temperature, atmospheric pressure, precipitation and relative humidity were obtained from the Daily Dataset of Ground Climate Data of China’s National Meteorological Information Center (NMIC) (https://data.cma.cn), which includes the observations of 699 base stations in the whole country (Supplementary Fig. S3).

Hourly pollutant concentration data and daily meteorological data were accumulated as monthly values. We defined March–May as spring, June–August as Summer, September–November as Autumn, and December-February as winter. To investigate the relationship between pollutant concentration and meteorological conditions at the site scale, we used the 896 air quality observation stations as reference station and applied Kriging interpolation technology to spatially interpolate meteorological elements, and then used the method of the Extract Values to Points of spatial analysis in Geographic Information System to extract the meteorological parameter value of each station. The maximum distance between meteorological station and air quality station was set to five km when performing Kriging interpolation. In the end, the monthly data sets of pollutant concentration and meteorological elements from 896 stations nationwide from June 2014 to February 2019 were obtained.

Followed a previous study^55^, we used the slope of the pollutant concentration and meteorological elements at each site from 2015 to 2018 to reflect the trend of pollutant concentration and meteorological elements. A positive slope indicates that pollutant concentration and meteorological elements are increasing, and vice versa. The slope is negative, and the larger its absolute value is, the faster the decline is. Partial correlation, Pearson’s correlation, MLR model and panel data model were applied to explore the nexus between pollutant concentration and meteorological parameters. Partial correlation analysis was applied to investigate the relationship between meteorological elements and pollutant concentrations. Pearson correlation analysis was used to detect the relationship between meteorological elements and pollutant concentration at each observation site. The statistically significant positive, negative, and uncorrelated correlations between the two variables at each site were shown in red, blue, and white, respectively. The MLR model was used to assess the impact of meteorological conditions on air pollutant concentration at each station. The MLR was estimated by OLS method. The significance of the MLR model was test by *F*-test, and that of independent variable was tested by student’s *t*-test. The MLR model is expressed as follows:

$${\text{y}}_{i} = {\text{b}}_{0} + {\text{b}}_{1} x_{i1} + {\text{b}}_{2} x_{i2} + {\text{b}}_{3} x_{i3} + {\text{b}}_{4} x_{i4} + {\text{b}}_{5} x_{i5} + {\upvarepsilon }$$

where *i* = 1, 2, 3, …., 896. *y* is dependent variable, i.e., concentration of six air pollutants; $x_{i}$ is independent variable, and $x_{1}$, $x_{2}$, $x_{3}$, $x_{4}$ and $x_{5}$ are average wind speed, temperature, atmospheric pressure, precipitation and relative humidity, respectively.

The panel data model was further used to evaluate the dynamic impact of changes in meteorological conditions on the concentration of air pollutants. After testing, we used a fixed-effect model to estimate the parameters. The model can be expressed as:

$$Y_{it} = \alpha_{i} + X_{it} {\upbeta } + {\upvarepsilon }_{it}$$

where *i* = 1, 2, 3, ….., 896; *t* = 1,2,3,…..,57 (from June 2014 to February 2019, in total 57 months); $\alpha$ is the intercept term,${{ \upbeta }}$ is the estimated coefficient of each explanatory variable, and ${\upvarepsilon }$ is the error term; *X* is the meteorological conditions (wind speed, temperature, precipitation, relative humidity, atmospheric pressure), and *Y* is the concentration of pollutants (PM_2.5_, PM_10_, SO_2_, NO_2_, CO and O_3_).

## Supplementary information

Supplementary information.

## Data Availability

The data that support the findings of this study are available from the corresponding authors upon request.
