# Supplementary material for: Exploring the relationship between air pollution and meteorological conditions in China under environmental governance
Source: Sci Rep. 2020 Sep 3;10:14518. doi: 10.1038/s41598-020-71338-7 (PMC7471117; doi:10.1038/s41598-020-71338-7)
Supplement: Supplementary file 1 — Supplementary information. [file 41598_2020_71338_MOESM1_ESM.docx]

**Exploring the relationship between air pollution and meteorological conditions in China under environmental governance**

**Yansui Liu^1,2^, Yang Zhou^1,2^*, Jiaxin Lu^1^**

1 Institute of Geographic Sciences and Natural Resources Research, Chinese Academy of Sciences, Beijing 100101, China

2 Key Laboratory of Regional Sustainable Development Modeling, Chinese Academy of Sciences, Beijing 100101, China

*Corresponding author at: Institute of Geographic Sciences and Natural Resources Research, Chinese Academy of Sciences, 11A Datun Road, Chaoyang District, Beijing 100101, China.

Tel.: +86 10 64889034

E-mail address: zhouyang@igsnrr.ac.cn (Y. Zhou)

**Supplement Information (SI):**

**Supplementary Table S1**. Partial correlation coefficients between air pollutant concentration and meteorological conditions in four seasons in China between 2014 and 2019.

| **Control variable** | **Variable** | **PM_2.5_** | | | | **PM_10_** | | | |
| --- | --- | --- | --- | --- | --- | --- | --- | --- | --- |
|  |  | **SP** | **SU** | **AU** | **WI** | **SP** | **SU** | **AU** | **WI** |
| **WS** | **Pre** | -0.369*** | -0.29*** | -0.422*** | -0.369*** | -0.588*** | -0.473*** | -0.565*** | -0.492*** |
|  | **AP** | 0.181*** | 0.159*** | 0.202*** | 0.123*** | -0.128*** | -0.088** | -0.048 | -0.043 |
|  | **Tem** | -0.152*** | 0.107*** | -0.323*** | -0.295*** | -0.367*** | -0.100** | -0.464*** | -0.379*** |
|  | **RH** | -0.287*** | -0.166*** | -0.251*** | -0.311*** | -0.599*** | -0.443*** | -0.445*** | -0.472*** |
|  |  | **SO_2_** | | | | **CO** | | | |
| **WS** | **Pre** | -0.23*** | -0.112*** | -0.325*** | -0.492*** | -0.260*** | -0.212*** | -0.417*** | -0.511*** |
|  | **AP** | -0.059* | -0.077** | -0.086*** | -0.127*** | -0.030 | -0.022 | -0.099** | -0.155*** |
|  | **Tem** | -0.120*** | -0.060* | -0.280*** | -0.497*** | -0.062* | -0.002 | -0.310*** | -0.385*** |
|  | **RH** | -0.296*** | -0.103** | -0.314*** | -0.582*** | -0.294*** | -0.244*** | -0.384*** | -0.59*** |
|  |  | **NO_2_** | | | | **O_3_** | | | |
| **WS** | **Pre** | -0.102** | -0.166*** | -0.274*** | -0.179*** | -0.319*** | -0.45*** | 0.299*** | 0.261*** |
|  | **AP** | 0.149*** | -0.024 | 0.076** | 0.116*** | -0.010 | 0.009 | 0.296*** | -0.043 |
|  | **Tem** | -0.063* | -0.053 | -0.264*** | -0.218*** | -0.219*** | -0.093** | 0.492*** | 0.324*** |
|  | **RH** | -0.107*** | -0.198*** | -0.219*** | -0.272*** | -0.281*** | -0.397*** | 0.208*** | 0.171*** |
|  |  | **PM2.5** | | | | **PM10** | | | |
| **PRE** | **WS** | -0.163*** | 0.042 | -0.24*** | -0.191*** | -0.142*** | 0.076** | -0.219*** | -0.165*** |
|  | **AP** | 0.326*** | 0.317*** | 0.35*** | 0.297*** | 0.084** | 0.138*** | 0.14*** | 0.174*** |
|  | **Tem** | 0.165*** | 0.36*** | 0.063* | -0.014 | 0.061* | 0.251*** | 0.018 | -0.016 |
|  | **RH** | 0.042 | 0.118*** | 0.276*** | 0.006 | -0.237*** | -0.109*** | 0.139*** | -0.119*** |
|  |  | **SO_2_** | | | | **CO** | | | |
| **PRE** | **WS** | 0.055 | 0.032 | -0.033 | 0.074** | -0.141*** | -0.061* | -0.217*** | -0.074** |
|  | **AP** | 0.034 | -0.028 | 0.017 | 0.079** | 0.047 | 0.057* | 0.013 | 0.049 |
|  | **Tem** | 0.006 | 0.007 | -0.021 | -0.248*** | 0.179*** | 0.161*** | 0.078** | -0.021 |
|  | **RH** | -0.196*** | -0.017 | -0.063* | -0.374*** | -0.131*** | -0.136*** | 0.014 | -0.325*** |
|  |  | **NO_2_** | | | | **O_3_** | | | |
| **PRE** | **WS** | -0.100** | 0.046 | 0.159*** | 0.2*** | 0.082** | 0.138*** | 0.256*** | 0.175*** |
|  | **AP** | 0.181*** | 0.058* | -0.058* | -0.114*** | 0.128*** | 0.253*** | 0.244*** | -0.153*** |
|  | **Tem** | 0.042 | -0.110*** | 0.072** | -0.18*** | -0.058* | 0.233*** | 0.416*** | 0.167*** |
|  | **RH** | -0.031 | -0.006 | -0.152*** | -0.084** | -0.046 | -0.047 | -0.166*** | -0.088** |
|  |  | **PM_2.5_** | | | | **PM_10_** | | | |
| **AP** | **WS** | 0.043 | 0.132*** | -0.183*** | -0.155*** | 0.196*** | 0.228*** | -0.104** | -0.112*** |
|  | **Pre** | -0.426*** | -0.410*** | -0.478*** | -0.436*** | -0.593*** | -0.518*** | -0.553*** | -0.503*** |
|  | **Tem** | -0.23*** | -0.091** | -0.416*** | -0.304*** | -0.388*** | -0.143*** | -0.46*** | -0.353*** |
|  | **RH** | -0.384*** | -0.322 | -0.267*** | -0.334*** | -0.617*** | -0.498*** | -0.392*** | -0.446*** |
|  |  | **SO_2_** | | | | **CO** | | | |
| **AP** | **WS** | 0.188*** | 0.075** | 0.020 | 0.098** | -0.010 | 0.009 | -0.138*** | -0.030 |
|  | **Pre** | -0.286*** | -0.110*** | -0.313*** | -0.487*** | -0.224*** | -0.210*** | -0.375*** | -0.491*** |
|  | **Tem** | -0.200*** | -0.029 | -0.269*** | -0.492*** | -0.035 | 0.020 | -0.260*** | -0.351*** |
|  | **RH** | -0.352*** | -0.088 | -0.292*** | -0.586*** | -0.267*** | -0.271*** | -0.299*** | -0.554*** |
|  |  | **NO_2_** | | | | **O_3_** | | | |
| **AP** | **WS** | -0.050 | 0.050** | -0.116*** | -0.072** | 0.264*** | 0.270*** | 0.195*** | 0.152*** |
|  | **Pre** | -0.118*** | -0.178*** | -0.29*** | -0.237*** | -0.415*** | -0.543*** | 0.183*** | 0.285*** |
|  | **Tem** | -0.080** | -0.073** | -0.301*** | -0.241*** | -0.346*** | -0.265*** | 0.361*** | 0.311*** |
|  | **RH** | -0.146*** | -0.226*** | -0.208*** | -0.315*** | -0.391*** | -0.508*** | 0.030 | 0.156*** |
|  |  | **PM_2.5_** | | | | **PM_10_** | | | |
| **Tem** | **WS** | -0.065* | 0.160*** | -0.224*** | -0.209*** | -0.065* | 0.195*** | -0.201*** | -0.187*** |
|  | **Pre** | -0.346*** | -0.462*** | -0.280*** | -0.247*** | -0.484*** | -0.538*** | -0.355*** | -0.350*** |
|  | **AP** | 0.254*** | 0.174*** | 0.360*** | 0.200*** | 0.024 | 0.062* | 0.166*** | 0.050 |
|  | **RH** | -0.254*** | -0.27*** | 0.057* | -0.122*** | -0.512*** | -0.462*** | -0.092** | -0.279*** |
|  |  | **SO_2_** | | | | **CO** | | | |
| **Tem** | **WS** | 0.081** | 0.054 | -0.033 | 0.012 | -0.045 | 0.007 | -0.200*** | -0.103** |
|  | **Pre** | -0.206*** | -0.105** | -0.173*** | -0.225*** | -0.278*** | -0.257*** | -0.291*** | -0.370*** |
|  | **AP** | 0.015 | -0.025 | 0.041 | 0.001 | -0.014 | -0.030 | 0.014 | -0.068** |
|  | **RH** | -0.296*** | -0.086*** | -0.146*** | -0.389*** | -0.347*** | -0.289*** | -0.167*** | -0.458*** |
|  |  | **NO_2_** | | | | **O_3_** | | | |
| **Tem** | **WS** | -0.083** | 0.035 | -0.155*** | -0.110*** | 0.086** | 0.245*** | 0.316*** | 0.214*** |
|  | **Pre** | -0.072** | -0.171*** | -0.100** | -0.043 | -0.245*** | -0.521*** | -0.206*** | 0.060* |
|  | **AP** | 0.170*** | 0.043 | 0.196*** | 0.176*** | 0.119*** | 0.221*** | 0.141*** | -0.124*** |
|  | **RH** | -0.087** | -0.203*** | 0.017 | -0.154*** | -0.184*** | -0.410*** | -0.358*** | -0.089** |
|  |  | **PM_2.5_** | | | | **PM_10_** | | | |
| **RH** | **WS** | -0.108*** | 0.112*** | -0.237*** | -0.23*** | -0.125*** | 0.169*** | -0.259*** | -0.242*** |
|  | **Pre** | -0.214*** | -0.285*** | -0.434*** | -0.246*** | -0.180*** | -0.260*** | -0.430*** | -0.265*** |
|  | **AP** | 0.332*** | 0.312*** | 0.267*** | 0.245*** | 0.167*** | 0.221*** | 0.085** | 0.140*** |
|  | **Tem** | 0.107*** | 0.211*** | -0.232*** | -0.120*** | 0.127*** | 0.148*** | -0.240*** | -0.098** |
|  |  | **SO_2_** | | | | **CO** | | | |
| **RH** | **WS** | 0.034 | 0.053 | -0.087** | -0.051 | -0.151*** | -0.033 | -0.268*** | -0.203*** |
|  | **Pre** | 0.014 | -0.065* | -0.135*** | -0.103** | -0.003 | -0.017 | -0.237*** | -0.152*** |
|  | **AP** | 0.092** | -0.017 | 0.018 | 0.124*** | 0.087** | 0.127*** | -0.003 | 0.074** |
|  | **Tem** | 0.095** | -0.014 | -0.077*** | -0.188*** | 0.247*** | 0.163*** | -0.063* | 0.004 |
|  |  | **NO_2_** | | | | **O_3_** | | | |
| **RH** | **WS** | -0.100** | 0.016*** | -0.174*** | -0.137*** | 0.113*** | 0.226*** | 0.264*** | 0.189*** |
|  | **Pre** | -0.004 | -0.018 | -0.202*** | 0.019 | -0.179*** | -0.294*** | 0.280*** | 0.229*** |
|  | **AP** | 0.198*** | 0.102** | 0.135*** | 0.231*** | 0.155*** | 0.314*** | 0.280*** | -0.098** |
|  | **Tem** | 0.057* | 0.061* | -0.172*** | -0.052 | -0.081** | 0.109*** | 0.535*** | 0.275*** |

Notes: Obs. is 896. WS, Pre, RH, Tem and AP indicate annual average value of wind speed, precipitation, relatively humidity, temperature and atmosphere pressure, respectively. SP, SU, AU and WI represent Spring, Summer, Autumn and Winter, respectively. *, ** and *** indicate statistical significance at the 10%, 5% and 1% levels, respectively.

**Supplementary Fig. S1.** Correlation between air pollutant concentration and meteorological conditions at the site scale.


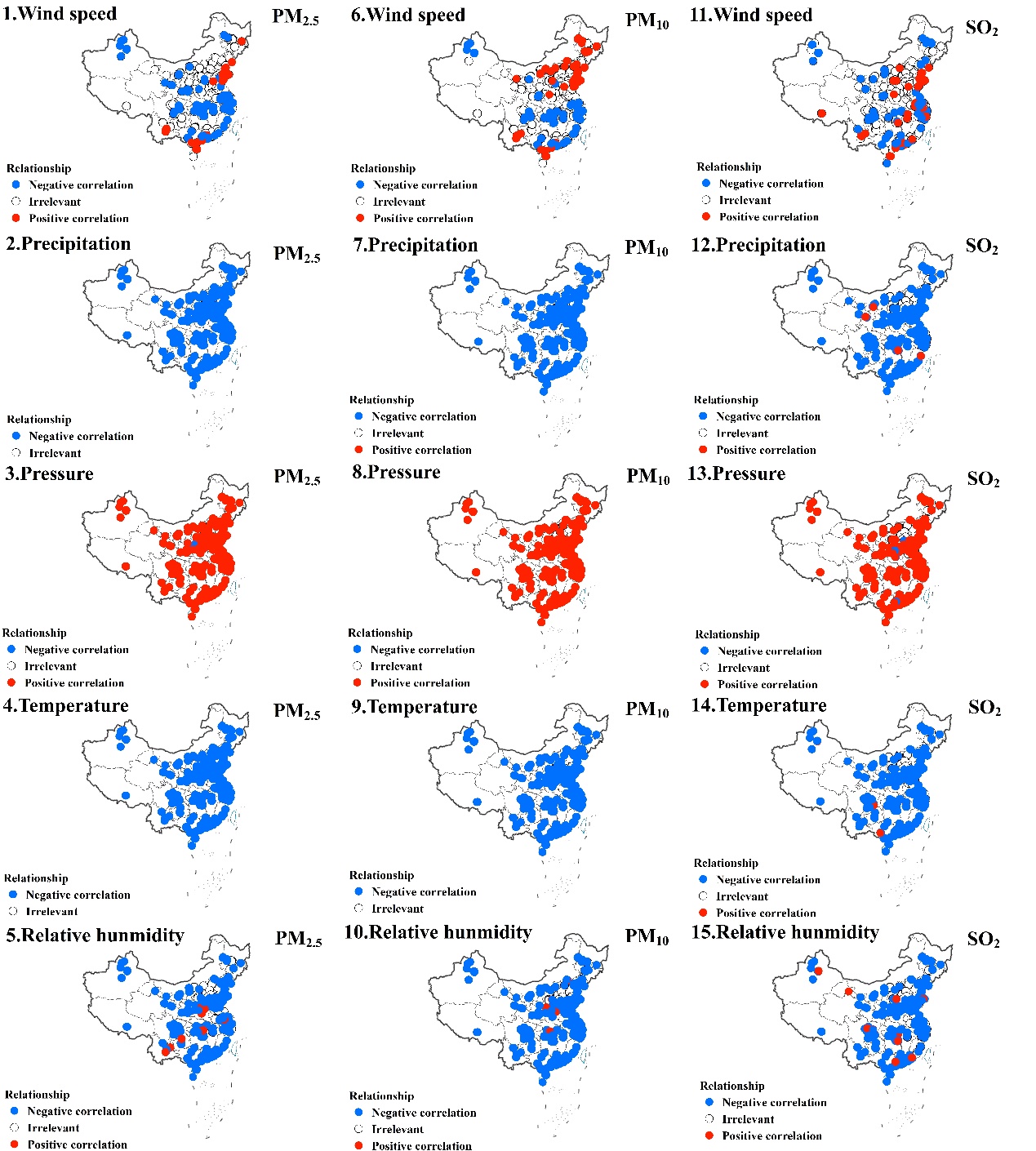


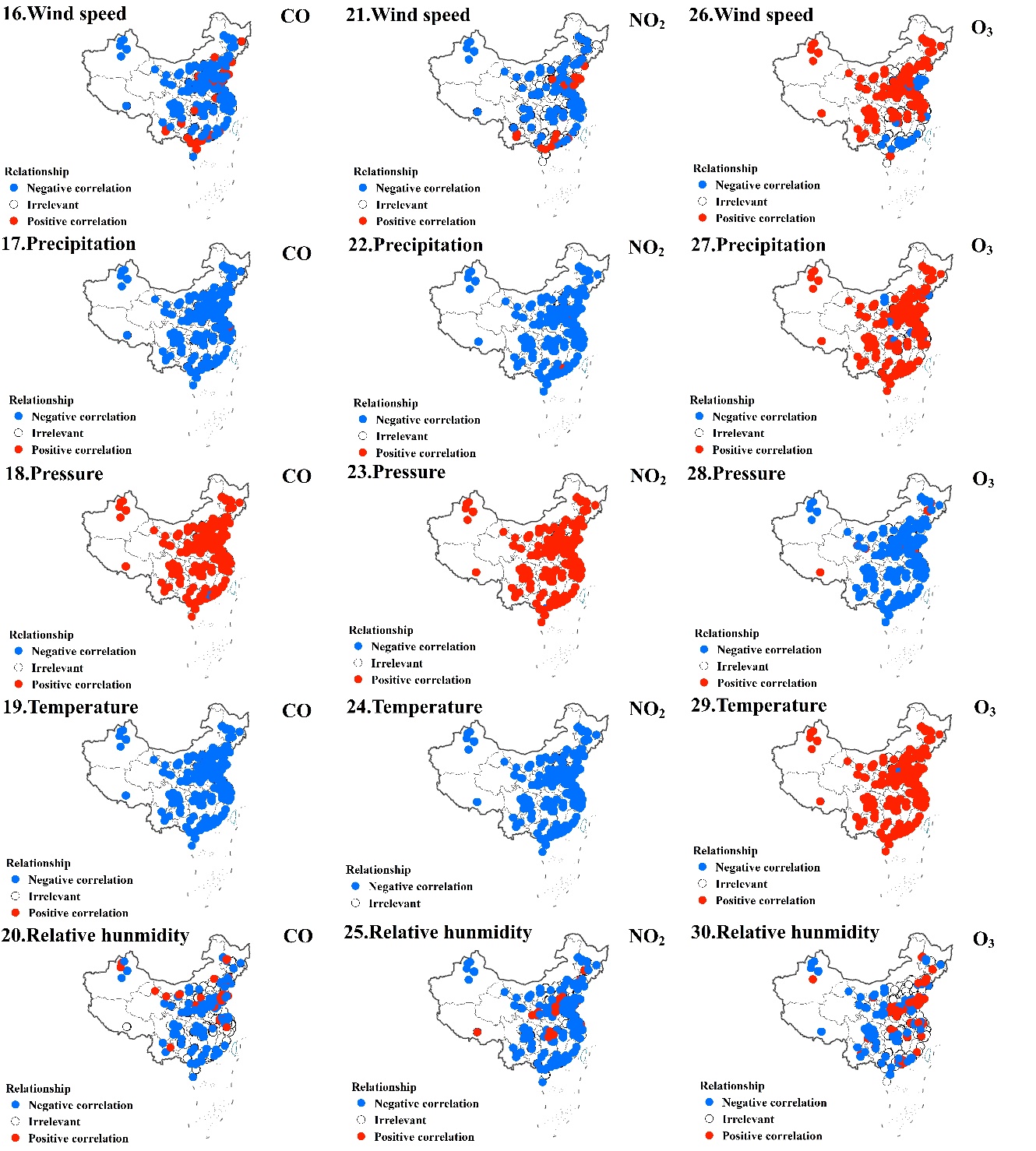


**Fig. S1.** Correlation between annual average pollutant concentration and weather conditions at 896 stations nationwide from June 2014 to February 2019. (Notes: The map was generated using ESRI’s ArcGIS 10.2 (<http://desktop.arcgis.com/en/arcmap>); Negative/positive correlations are based on the judgement at the 10% level or higher level. Irrelevant means that the correlation between the two variables is not significant at the 10% level or higher level. In the Figure, 1~5 (vertical), 6~10, 11~15, 16~20, 21~25 and 26~30 represent the concentration of PM_2.5_, PM_10_, SO_2_, CO, NO_2_ and O_3_, respectively.).

**Supplementary Fig. S2**. The correlation between pollutant concentrations at the site level.


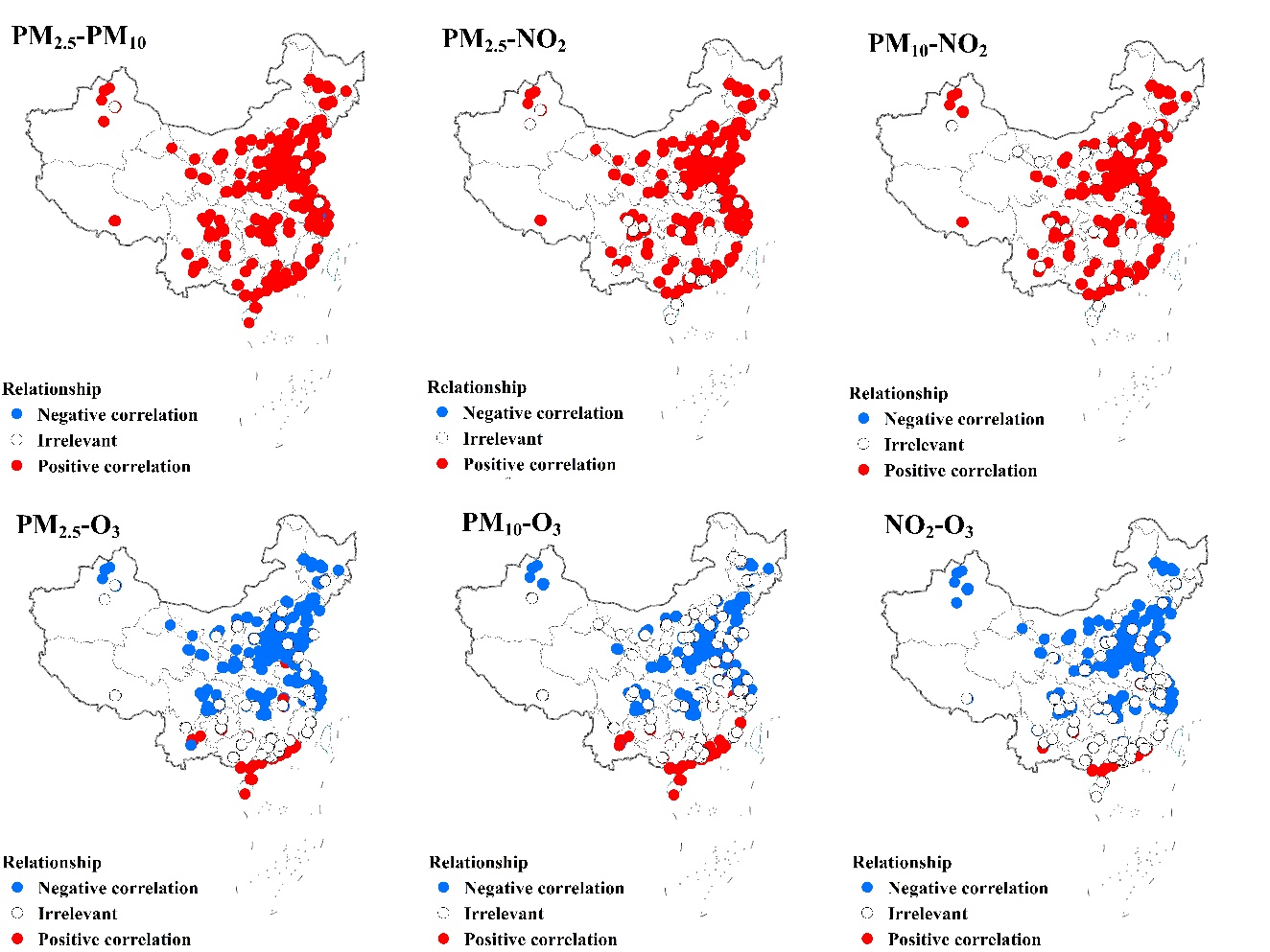


**Fig. S2**. The correlation between pollutant concentrations at the site level (Note: The map was generated using ESRI’s ArcGIS 10.2 (http://desktop.arcgis.com/en/arcmap/).

**Supplementary Fig. S3.** Spatial distribution of pollutant monitoring stations and meteorological stations in China.


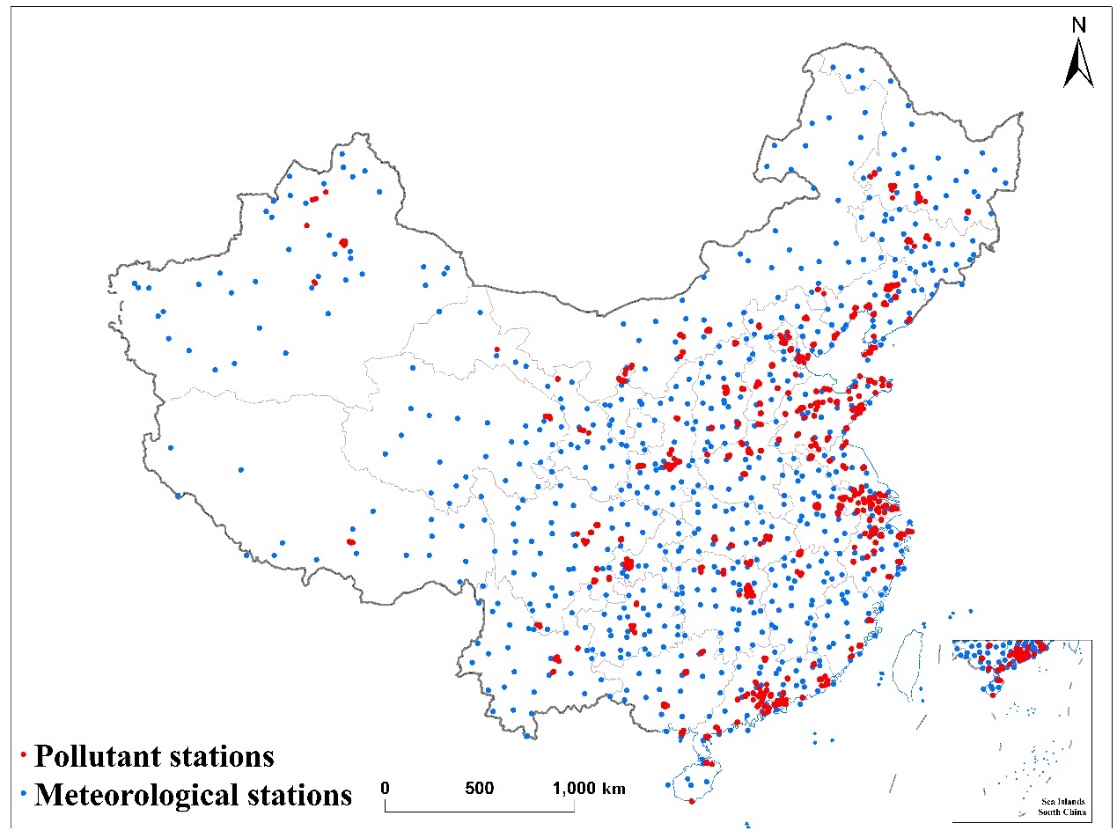


**Fig. S3.** Spatial distribution of pollutant monitoring stations and meteorological stations in China (Note: The map was generated using ESRI’s ArcGIS 10.2 (http://desktop.arcgis.com/en/arcmap/).
